# Aortic 4D flow: quantifying the effects of contrast and field strength at 1.5 T, 3T and 7T

**DOI:** 10.1186/1532-429X-16-S1-P169

**Published:** 2014-01-16

**Authors:** Aaron T Hess, Malenka M Bissell, Andrew Lewis, Ntobeko A Ntusi, Jane M Francis, Saul G Myerson, Stefan Neubauer, Matthew D Robson

**Affiliations:** 1 University of Oxford Centre for Clinical Magnetic Resonance Research, Oxford, UK

## Background

4D flow is a promising new method for assessment of aortic pathology, but is limited by signal-to-noise ratio (SNR) leading to long acquisition times. Higher field strength may prove a solution. SNR in aortic 4D flow increases at 3T versus 1.5T (Strecker et al, JMRI 2012) and further by adding a contrast agent (Bock et al, MRM 2010). This work extends this comparison to human 7 Tesla and quantifies the field strength dependent effects of contrast agents.

## Methods

Four healthy male volunteers were scanned six times: both with and without contrast (MultiHance, Braco, Milano, Italy) at each field 1.5 T, 3 T, and 7 T. All scans were acquired within four weeks and on Siemens scanners. Identical protocols were used, TR/TE 4.33/2.5 ms, temporal resolution 52 ms, bandwidth 1502 Hz/pixel, matrix 192 × 124 × 24, field of view 384 × 310 × 60 mm^3^, resolution 2.0 × 2.5 × 2.5 mm^3^, flip angle 7°, segmentation 3, GRAPPA 2, VENC 150 cm/s. At 7 T no RF spoiling was employed and a flip angle post B1 shimming ranging from 5° to 7° through the aorta. 7 T scans employed dynamic B1 shimming alternating between navigator and aorta specific shim. SNR was calculated by taking the difference of two symmetrically flow-encoded in one direction magnitude images (enc1 and enc3). SNR(**r**) = mean(enc1(**r**, t) + enc3(**r**, t))/√2 (std(enc1(**r**, t) - enc3(**r**, t))), **r **is the spatial coordinate and t are the temporal frames during diastole. SNR was assessed in the descending aorta over a 40 × 7.5 mm^2 ^× aorta diameter ROI centered 60 mm below the midpoint of the aortic arch.

## Results

SNR for each of the six scans for each subject are plotted and a set of stream lines seeded in the LV (7 T data) are shown. The mean ± SD increase in SNR due to contrast agent is 1.8 ± 0.2, 1.8 ± 0.5 and 1.4 ± 0.2 for 1.5 T, 3 T and 7 T respectively. The mean ± SD increase in SNR due to field strength without and with the contrast agent is 1.8 ± 0.4 and 1.7 ± 0.1 for 1.5 T to 3 T and 2.1 ± 0.7 and 1.7 ± 0.4 for 3 T to 7 T. The average difference in peak net flow rate at the same location in descending aorta at 7 T compared to 3 T was 7 ± 7 ml/s. Of interest is that increases in SNR by stepping up in field strength are comparable to the increase from contrast.

## Conclusions

4D aortic flow is feasible at 7 Tesla and yields substantial SNR improvements over lower field strengths. Future work will exploit this higher SNR to explore improved spatial and/or temporal resolution.

## Funding

Medical Research Council (UK) and British Heart Foundation.

**Figure 1:**
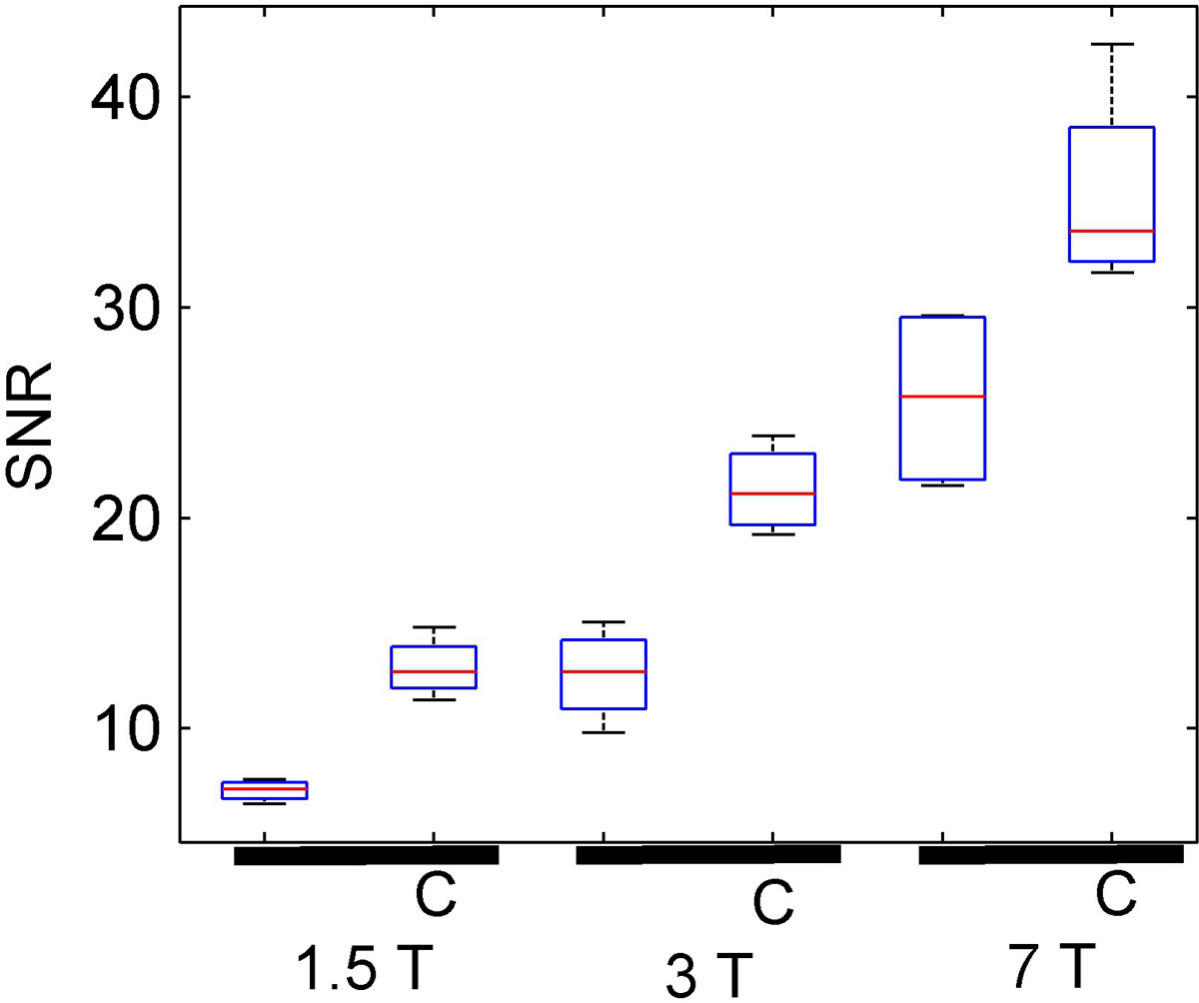
**Box and whisker plots of SNR at each field strength in the descending aorta at 1.5 T, 3 T, and 7 T both without and with a contrast agent (C)**.

**Figure 2:**
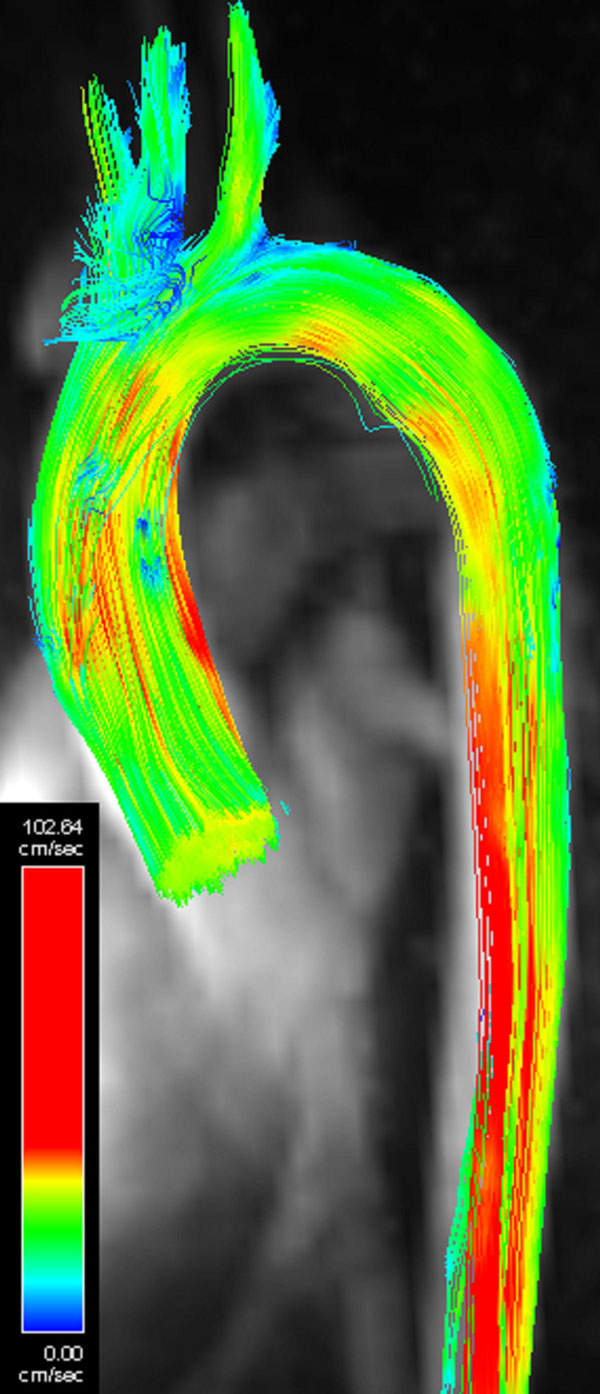
**Streamlines generated from a 7 T scan with contrast using software from Siemens and by seeding the streamlines in the left ventricle at 155 ms after the ECG R wave**.

